# Evidence, rationality, and ignorance: Agnotological issues in COVID-19 science

**DOI:** 10.1590/0037-8682-0475-2020

**Published:** 2020-09-21

**Authors:** Carlos Magno Castelo Branco Fortaleza

**Affiliations:** 1 Universidade Estadual Paulista, Faculdade de Medicina, Câmpus de Botucatu, Departamento de doenças infecciosas, Botucatu, SP, Brasil.

**Keywords:** Coronavirus disease, Epidemiology, Knowledge, Philosophy

## Abstract

Two decades ago, Robert Proctor coined the term *agnotology* to refer to the study of ignorance that stems from scientific research. Amid the coronavirus disease pandemic, the world is witnessing the greatest natural experiment ever, and countries have adopted different response strategies. An evaluation of the effectiveness of different policies will play a valuable role in preparing for future public health emergencies. However, controversial issues such as the timing and pathways of viral emergence, the effectiveness of social distancing and lockdown strategies, and the use of antimalarial drugs as therapy have still not been fully resolved. This serves as a fertile breeding ground for agnotological strategies, whereby scientific studies are deliberately or unintentionally designed to create distractions or draw conclusions that are not supported by research findings. Researchers, public health authorities, and healthcare workers should be equipped to identify such agnotological strategies, distinguish them from scientific fraud, and avoid drawing misleading inferences based on an irrational adherence to hypotheses and a lack of criticism of implausible results.

## INTRODUCTION

In his famous essay titled, *On the Sources of Knowledge and Ignorance* ^1^, the Austrian-British philosopher Karl Popper (1902-1994) has discussed a series of fundamental issues that address the objectivity of scientific knowledge. It may surprise an orthodox practitioner of evidence-based medicine (EBM) to learn that, in accordance with a tradition that dates back to David Hume (1711-1776), Popper rejected induction, which refers to the notion that a series of observations and experiments (i.e., evidence) allows one to draw inferences about general scientific laws or generate “recommendations.” Popper’s views can be summarized as follows: “there are several sources of knowledge, but science progresses blindly toward the truth rather by eliminating errors (by continuous criticism and empirical research) than from gathering cumulative evidences.” Despite major ideological differences, some of the greatest 20^th^ century philosophers of science (Popper^2^, Kuhn^3^, Lakatos^4^, and Feyerabend^5^) prioritized rationality over the accumulation of evidence. The Hippocratic aphorism, “Life is short, and art long, opportunity fleeting, experimentations perilous, and judgment difficult,” is surprisingly consistent with these views^6^. The mythic father of medicine warns us that evidence is often misleading and that experiments can lead to ignorance.

How do epistemological disputes concern scientific responses to the coronavirus disease (COVID-19) pandemic? At this juncture, we must recall Robert Proctor’s (born 1954) concept of *agnotology*, which refers to the study of the production of ignorance (especially through scientific research)^7^. Proctor was interested in ignorance as an active construct, which refers to the deliberate use of scientific research to distract or distort public attention. This is much more sophisticated than scientific fraud. Active construct agnotological strategies typically include two basic strategies: (i) a continuous call for more evidence and (ii) a shift in emphasis toward competing causal chains. Both strategies have been widely used by the tobacco industry (e.g., funding studies on other carcinogenic substances or microorganisms) and climate deniers^8^. Agnotological phenomena can also emerge as a passive construct. *Bona fide* scientists may focus on one aspect of research so intensively that their methods lack attunement to other relevant information^9^. Finally, some authors have theorized about a virtuous agnotological behavior (i.e., a deliberate value-laden ethical decision to not conduct research on certain topics; e.g., the association between gender and intelligence, appropriate human cloning methods)^10^. In the following sections, I delineate how the agnotological issues raised in “emergency science”^11^ can support responses to the COVID-19 pandemic.

## COVID-19 SCIENCE

From a researcher’s perspective, the world is witnessing the greatest natural experiment ever. Different countries have adopted varied strategies to prepare for and respond to the COVID-19 pandemic^12^. The catastrophic impact of the pandemic on both health and economics has fueled politically charged discussions on social restriction and lockdown policies^13^. This issue requires much research, but it is very unlikely that any *evidence* (as used by the Cochrane Collaboration) will emerge in the near future^11^. Further investigation into this issue lies beyond the scope of this article. However, a rational and critical attitude is highly valuable during times of uncertainty. In the following sections, I provide selected examples of the use of agnotological strategies in COVID-19 science. I assume that no fraud was involved in data collection or analysis in these studies.

## IGNORANCE AS AN ACTIVE CONSTRUCT

In an ecological study, Izoulet^14^compared COVID-19 daily deaths in countries that had recommended the use of antimalarial drugs (i.e., chloroquine and hydroxychloroquine) as therapy during the early phases of the epidemic and those that had not issued such recommendations. The complex methodology entailed an analysis of the dynamics of deaths between the day of the 3^rd^ death and the following 10 days. These datasets and Box-Jenkins models^15^ were used to make predictions about mortality trends in different countries. “Unsurprisingly,” the author has stated, “[the] models predict a stabilization of the number of deaths for the group of countries using chloroquine and a large increase for the group of countries not using it.” The conclusion also carries a grandiloquent tone: “the differences in dynamics is so striking that we believe that the urgency context commands presenting this ecological study before delving into further analysis.” In other words, the author has recommended the urgent adoption of antimalarial drugs as therapy for COVID-19 based on a self-admittedly non-in-depth preliminary analysis of forecasting trends derived from aggregate data from different countries.

Several factors underscore the active agnotological strategies used by this author. Although scientific documents typically carry a dispassionate tone, enthusiastic words such as “excellent model,” “very effective,” and “highly predictive” abound in the article. The article also contains a “study limitations” section, which subtly addresses the potential role of confounding variables and the limits of the forecasting models. However, there is not even a single reference to the ecological fallacy (i.e., the possibility of drawing incorrect inferences about individuals based on aggregate data drawn from the population to which they belong). This is not a minor issue. Even if ecological studies are an appropriate means of assessing collective public policies^16^, they do not provide any rational basis for an individual clinical approach. According to a recent review, “the only way to overcome such bias, while avoiding uncheckable assumptions concerning the missing information, is to supplement the ecologic with individual-level information”^17^.

The debate surrounding the effectiveness of chloroquine and hydroxychloroquine still remains contentious and politically charged, even though randomized controlled trials (RCTs) have yielded no strong evidence of their benefits, and their severe adverse effects have caused agencies (e.g., the United States Food and Drug Administration) to contraindicate their use^18^. Thus, at present, verbal enthusiasm and the contention that the urgency of the response validates the use of antimalarial drugs as therapy for COVID-19 (based on aggregate data forecasting) are nothing but agnotological trickery.

## IGNORANCE AS A PASSIVE CONSTRUCT

At present, it is assumed that severe acute respiratory syndrome coronavirus 2 (SARS-CoV-2) emerged in Hubei province in China in late 2019^19^. However, according to a recent preprint posted on medRxiv, the virus was present in sewage samples collected from Florianópolis, South Brazil, and stored since November 2019^20^. Sewage collection and storage methods and laboratory techniques have been clearly described. Results are presented for serial sewage collections, and they are indicative of substantial viral loads in November and December 2019 as well as February and March 2020.

My objective is not to refute these findings but to articulate why they should be interpreted with due caution. First, and contrary to the previous example, they are presented concisely (i.e., similar to a “short communication” paper). Nevertheless, it has received substantial attention from the national press and has been considered as a potential source of conspiracy theories (https://brazilian.report/power/2020/07/08/new-study-gives-china-opportunity-blame-brazil-covid-19-virus/). If groundbreaking scientific information that can change our entire understanding of the epidemiology of COVID-19 exists, it deserves much more attention from the international scientific community than it has actually received. The authors must strengthen their findings if they wish to address justifiable widespread skepticism. As researchers, they deserve the benefit of the doubt. Nevertheless, the agnotological issues involved in this study pertain to semantics. The authors have stated that their results “show that SARS-CoV-2 has been circulating in Brazil since late November 2019.” *Circulate* is an ambiguous term that is not indexed in the International Epidemiological Association’s *Dictionary of Epidemiology* ^21^. It appears to imply that there was active community transmission in South Brazil in 2019. This conclusion contradicts surveillance data, which point toward the late epidemic peak in the Southern Region of Brazil, as well as the findings of a national seroprevalence survey, which identified few cases in this region^22^. In a country where responses to COVID-19 lead to serious political disputes^23^, a report on the early detection of SARS-CoV-2 positivity in sewage samples cannot be readily translated as evidence of *circulation*. Even if the findings prove to be true, the epidemiological inferences embedded in the conclusion will remain agnotological.

## VIRTUOUS IGNORANCE

Microorganisms and diseases are traditionally named after the place in which they are first reported. Examples include Zika^24^, Ebola^25^, and Rocky Mountain Spotted Fever^26^. The issue of stigma against local residents was raised when a novel hantavirus was named Muerto Canyon after it was discovered in a Navajo Reservation with the same name. Public objection led to a change in nomenclature, and it is now known as the “Sin Nombre” (Spanish term for “nameless”) virus^27^.

The emergence of SARS-CoV-2 in China created a wave of prejudice all over social media^28^. Terms such as “Chinese virus” and “Kung-Flu” have been accompanied by racist comments and criticisms of the eating habits of Chinese individuals. This was a major factor that influenced the World Health Organization’s decisions regarding the naming of the virus and disease^29^. This is an example of a virtuous agnotological strategy. It is not that we do not want to know how the virus emerged; indeed, this fact provides crucial information that can be used to prevent future pandemics. However, health authorities and researchers who adhered to ethical standards decided that they did not want to be reminded of the origin of the virus (and, thus, contribute to prejudice) each and every time the disease is discussed. If science ought to play a role in promoting peace, harmony, and human values, then partially forgetting information (when it is not immediately relevant) can be considered as a virtuous action^30^.

## RATIONALISM, EMPIRICISM, AND EBM

Rationalism (i.e., the ideology that reason is the chief source and test of knowledge^31^) dates back to the Greek philosophers. However, the fundamentals of modern science were born in the 16^th^ Century, when Francis Bacon (1561-1626) and others posited that only knowledge grounded in observation and/or experimentation is valid^32^. Medicine has its roots in prehistory and is far older than what we now call *science*. The incorporation of the concepts of empiricism into medical practice (i.e., the birth of *scientific medicine*) can be traced back to the late 18^th^ or early 19^th^ century^33^.

The apex of the influence of empiricism emerged when Austin Bradford Hill (1897-1991) introduced RCTs, based on Ronald Fisher’s (1890-1962) prior experience with randomizing territories for agronomic research^34^. Even though Hill was particularly concerned with validating inferences drawn based on cohort studies, his criteria for causality in health sciences (which are still used today) prioritize experimentation (i.e., RCTs) over observation (e.g., cohort and case-control studies)^35^. EBM is a late radical development that followed this trend^33^.

Except for research on therapeutic options and vaccines, the EBM hierarchy regarding the strength of evidence is not readily applicable to the COVID-19 response^11^ ^,^ ^36^. This does not imply that any preventive policy is irrational or founded upon weak scientific ground. Indeed, EBM has been criticized by modern philosophers of science^37^ ^,^ ^38^. The focus of these criticisms is not the empirical value of the evidence but the rigidity of the methodological hierarchy. Indeed, RCT subjects are often not representative of the members of the general population with the disease (or at risk, in the case of vaccines). In many cases, this leads to the paradox of high internal validity and poor external validity and, consequently, limits the generalizability of the findings to real-life settings^39^. In such cases, a strictly orthodox concept of evidence can render healthcare professionals vulnerable to error and ignorance. Thus, agnotological issues exist even within orthodox EBM value systems.

It is noteworthy that even some of Hill’s criteria for causality (consistency, plausibility, and consideration of alternative explanations)^35^ entail a rationalist filter for empirical findings. Further, when subjected to appropriate rational criticism (considering possible sources of bias), studies deemed “suboptimal” as per conventional EBM criteria (e.g., nonrandomized *quasi-*experimental studies, natural experiments, and even computational modeling) play a very valuable role in guiding public health policies^40^. 

## CALL FOR FURTHER EVIDENCE, AN IMPACT-CENTERED APPROACH, AND FALSE ADVERTISING

As mentioned earlier, calls for further evidence are a classic agnotological strategy used by the tobacco industry and climate deniers^7^ ^,^ ^8^ ^,^ ^30^. This strategy can be conceptualized as a two-way street. Consider the debate surrounding chloroquine and hydroxychloroquine use. One group claims that there is evidence to suggest that they are good therapeutic options, whereas another group claims that more evidence indicates that they are useless^41^. Carrier has proposed rules that can be used to escape from these kinds of puzzles and, at the same time, identify agnotological strategies^42^ ^,^ ^43^. The first rule is to shift evidence standards for potentially harmful hypotheses (“impact-centered approach”). Thus, if a novel therapeutic approach for SARS-CoV-2 infection is proposed, the requirements of the evidence should be higher in the face of the risk of adverse events. The second rule is “avoid false advertisements” (i.e., reports in which the inferences are much broader than what the findings suggest). This often stems from the extrapolation of *in vitro* or animal experimental findings to clinical practice among human beings. It is noteworthy that two of the aforementioned studies^14^ ^,^ ^20^ belong to this category.

## GOVERNMENTAL AGNOTOLOGICAL STRATEGIES

The promotion of scientific ignorance for political purposes has been studied extensively. Most authors have examined the issue of climate denialism in the United States and Europe^44^. However, the analyzes took a new direction in view of the unrestricted and uncritical adherence to chloroquine/hydroxychloroquine therapy of influential leaders, such and the presidents of the United States and Brazil^23^ ^,^ ^25^ ^,^ ^45^. These positions are based on the following pseudo-question: *Is early treatment an option that can prevent the recession caused by social distancing protocols?* The fact that well-designed studies that compare the two strategies are both ethically and operationally unfeasible in the short term has facilitated the emergence of several agnotological strategies based on surveillance data or modeling. For example, on the Brazilian COVID-19 surveillance homepage (https://covid.saude.gov.br/), the number of cured individuals is prominently displayed in a large font in a green box, whereas the numbers of cases and deaths are discreetly presented in a smaller font. Even though it is rather obvious to epidemiologists that the high number of cured individuals is a direct consequence of the high number of cases, this message may not be easily comprehended by the general public. Therefore, emphasizing the number of cured persons instead of the number of reported cases is not an innocent decision. Instead, it exemplifies a governmental agnotological strategy during the pandemic era.

## FINAL REMARKS

This review aimed to introduce different agnotological strategies to researchers and healthcare workers so that they can identify active construct strategies (deliberate production of ignorance) and avoid passive construct strategies (*bona fide* errors based on a strong adherence to a hypothesis or a lack of criticism of implausible results). Virtuous ignorance is an ethical choice and must, therefore, be guided by human, social, and cultural values. In accordance with the second (but not less important) objective, I have underscored the limited value of evidence that has not been submitted to rational filters^11^ ^,^ ^38^ ^,^ ^39^ ^,^ ^45^. I have deliberately avoided a third focus area, namely, discussions on scientific fraud, data reliability, and articles retracted from prestigious peer-reviewed scientific journals. These are issues of great relevance to which interested readers should be directed^46^.

A network of mutual criticism that combines rationality and empiricism should be the basis of any scientific endeavor, including our response to the COVID-19 pandemic^2^ ^,^ ^4^ ^,^ ^12^. Thus, while epistemological considerations cannot be imposed as paralyzing or nihilistic arguments, the possibility of modifying public policies based on new findings guarantees that we are always looking for (and are perhaps close to) the right path^11^ ^,^ ^47^. The scientific debate must continue, but it must, as far as possible, be free from rigid ideas and political noise.
